# Identification of Potential Biomarkers for Psoriasis by DNA Methylation and Gene Expression Datasets

**DOI:** 10.3389/fgene.2021.722803

**Published:** 2021-08-26

**Authors:** Yong Liu, Shengnan Cui, Jiayi Sun, Xiaoning Yan, Dongran Han

**Affiliations:** ^1^School of Life Sciences, Beijing University of Chinese Medicine, Beijing, China; ^2^Department of Dermatology, Shaanxi Hospital of Chinese Medicine, Xi’an, China; ^3^Xiyuan Hospital, China Academy of Chinese Medical Sciences, Beijing, China

**Keywords:** psoriasis, DNA methylation, gene expression, AMPK signaling pathway, IRS1, ARHGEF10, RAI14

## Abstract

DNA methylation (DNAm) plays an important role in the pathogenesis of psoriasis through regulating mRNA expressions. This study aimed to identify hub genes regulated by DNAm as biomarkers of psoriasis. Psoriatic skin tissues gene expression and methylation datasets were downloaded from Gene Expression Omnibus (GEO) database. Subsequently, multiple computational approaches, including immune infiltration analysis, enrichment analysis, protein–protein interaction (PPI) network establishment, and machine learning algorithm analysis (lasso, random forest, and SVM-RFE), were performed to analyze the regulatory networks, to recognize hub genes, and to clarify the pathogenesis of psoriasis. Finally, the hypermethylated genes were used to immune cell infiltration analysis, which revealed that psoriasis skin tissues were mainly composed of activated dendritic cells, resting mast cells, T follicular helper cells (cTfh), etc. Differentially expressed-methylated genes (DEMGs) were identified and partitioned into four subgroups and the 97 significantly hypermethylated and downregulated (hyper-down) genes accounted for the highest proportion (47%). Hyper-down genes were mainly enriched in glucose homeostasis, AMP-activated protein kinase (AMPK) signaling pathway, lipid storage disease, partial lipodystrophy, and insulin resistance. Furthermore, insulin receptor substrate 1 (IRS1), Rho guanine nucleotide exchange factor 10 (ARHGEF10) and retinoic acid induced 14 (RAI14) were identified as potential targets. These findings provided new ideas for future studies of psoriasis on the occurrence and the molecular mechanisms.

## Introduction

Psoriasis is a chronic skin disease mediated by immune mechanisms that affect 2–3% population of the world, and the WHO considers psoriasis to be chronic, painful, non-infectious, incurable, disabling, and disfiguring [World Health Organization (WHO), 2014]. A higher prevalence of 4.6–4.7% in North America, whereas the prevalence in African and Asian populations is 0.4–0.7% (Perera et al., 2012; Parisi et al., 2013). Plaque psoriasis accounts for 80–90% of all cases, characterized by keratinocyte abnormal differentiation and hyperproliferation with a large number of inflammatory cell infiltration, which is the most common psoriasis subtype (Greb et al., 2016). It is a systemic inflammatory disease associated with hypertension, hyperlipidemia, metabolic syndrome, adverse cardiac events, etc., which will increase the incidence of malignant tumors, inflammatory arthritis, obesity, and other comorbidities (Lebwohl et al., 2014; Takeshita et al., 2017; Kaushik and Lebwohl, 2019). The pathogenesis is mainly T-cell-mediated immune dysfunction and is related to genetics and the environment (including infection, drugs, mental stress, and climate) (Armstrong and Read, 2020).

As we all know, epigenetics mediates the occurrence of autoimmune diseases and cancers mainly by regulating biological modification and cell differentiation and cycle (Zhang et al., 2013; Feinberg, 2018). DNA methylation (DNAm) is a type of epigenetic modification. Studies on the pathogenesis of many immune-related diseases (such as psoriasis) and tumors have shown that DNAm is a very important molecular mechanism (Zhang et al., 2013). Many studies had shown that epigenetic changes, including abnormal DNAm patterns, differentially methylated sites (DMSs), differentially methylated regions (DMRs), and histone modifications, were involved in psoriasis (Roberson et al., 2012; Zhou et al., 2016; Mazzone et al., 2019; Shao and Gudjonsson, 2020). Previous studies had shown that the DNAm level of skin samples from patients with psoriasis was positively correlated with the Psoriasis Area and Severity Index (PASI) score, and the levels of DNAm in skin lesions and peripheral blood monocytes (PBMCs) were significantly increased in patients with psoriasis (Chen et al., 2008; Zhang et al., 2010). Chen et al. (2016) identified the characteristics of human leukocyte antigen (HLA)-C hypermethylation in psoriasis skin lesions, which can be used as an epigenetic marker of psoriasis.

In this study, we integrated psoriasis DNAm datasets and gene expression datasets downloaded from the Gene Expression Omnibus (GEO) database to measure gene methylation levels. The proportion of immune cells with hypermethylated gene expression profiles in psoriatic and normal tissue samples was quantified using the CIBERSORT method. Machine learning algorithms are increasingly being used to screen gene targets, and we used this method to identify potential biomarkers of psoriasis, providing a certain research basis for further research on the pathogenesis of psoriasis.

## Materials and Methods

### Microarray Data Collection

**FIGURE 1:**
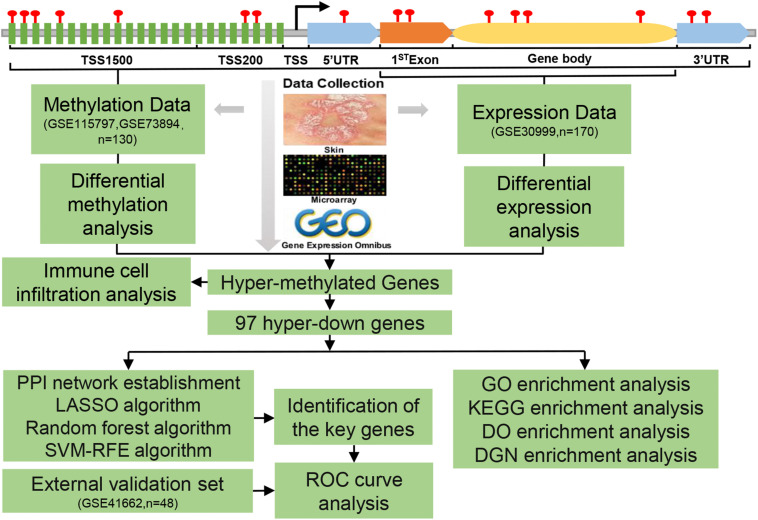
Flowchart of the analysis process.

The flowchart for this study was shown in Figure 1. The datasets of DNAm and mRNA expression profiles in psoriatic skin tissues and adjacent normal skin samples required for this study were obtained from the National Center for Biotechnology Information (NCBI), GEO database. We included datasets of the same platform to reduce the heterogeneity between different datasets. Only two DNAm datasets were up to the selection criteria, genome-wide DNAm profiling array (GSE115797), containing data from 48 samples with 24 paired tissues (Chandra et al., 2018), and genome-wide DNAm profiling array (GSE73894) including 82 samples with 41 paired tissues (Zhou et al., 2016; Shen et al., 2018), both datasets were generated by the platform GPL13534 (Illumina HumanMethylation450 BeadChip) from the United States.

Gene expression profiling array (GSE30999) (Suárez-Fariñas et al., 2012; Correa da Rosa et al., 2017) provided mRNA expression data from 170 skin biopsy samples with 85 psoriasis lesions and 85 matched non-lesional skin. A total of 48 samples with 24 psoriasis lesions and 24 matched non-lesional skin were obtained from the GSE41662 (Bigler et al., 2013) gene expression profiling, both datasets were generated by the platform GPL570 (Affymetrix Human Genome U133 Plus 2.0 Array) from the United States.

### Differential Gene Expression Analysis

The R package named “Limma” (Ritchie et al., 2015) was used to screen differentially expressed genes (DEGs) from the gene expression matrix, and the screening threshold was set as adj.*p*-value < 0.05 and |log_2_FC (fold change)| > 1. It should be noted that when multiple probes correspond to one gene, the average value of such genes should be calculated, probes corresponding to multiple genes and those without matching genes should be eliminated.

### Differential DNA Methylation Analysis

Illumina HumanMethylation450 BeadChip can detect 450,000 CpG sites, which cover the body (the area between the ATG start site and the stop codon), and 1st exon (the first exon of a gene), TSS1500 (Transcription start site 200–1,500 bases), TSS200 (Transcription start site 0–200 bases), 3′untranslated region (3′UTR) and 5′untranslated region (5′UTR) are widely used in DNAm studies.

Because of the two datasets based on the same platform GPL13534. Besides, the methylation chip datasets GSE73894 and GSE115797 had 48 and 82 samples, respectively, compared with the gene expression profile chip dataset GSE30999 (170 samples), the use of a small number of samples could affect the performance of statistical analysis and provide unreliable results. Therefore, we first integrated the two methylation chip datasets to significantly improve the number of samples. Given that the two datasets have different populations, we chose the ComBat method to remove the batch effect between the two datasets to reduce the potential heterogeneity (Leek et al., 2012). The ComBat method was used to normalize the beta values from different batches or platforms.

The methylation level of the gene was represented by the average beta value of CpG in different regions of the gene. The beta value matrix was analyzed by the R package named “limma” to screen differentially methylated CpGs (DMCs) sites, DMRs, and DMGs. The thresholds for judging difference were set as adj.*p*-value 0.05 and |log_2_FC (delta Beta)| > 0.05. The diagram drawn by the R package named “Rideogram” would be used to show the distribution of DMCs on different chromosomes (Hao et al., 2020), and the diagram drawn by the R package named “Upset” would be used to show the distribution of DMCs in different gene regions (Conway et al., 2017). The intersection of DEGs and DMGs was represented by DEMGs. The intersection of differentially methylated genes and DEGs (the threshold of differential analysis was adj.*p*-value < 0.05) was represented by DMEGs.

### Immune Infiltration Analysis

Through CIBERSORT^1^ algorithm to predict the immune cells associated with psoriasis DMEGs infiltration ratio, cluster analysis was performed according to the relative abundance of immune cells in different samples, and heatmap was drawn using the R package “pheatmap” to show the clustering results. Then, a box plot was used to show the difference of infiltration of different immune cells between psoriasis and the control group, which was plotted through the R package “ggplot2.” Finally, the correlation coefficient among immune cells was calculated with the R package “Corrplot,” and the relevant situation was plotted.

### Functional Enrichment Analysis

Gene ontology (GO) and Kyoto Encyclopedia of Genes and Genomes (KEGG) pathway enrichment analysis were performed by the R package named “clusterProfiler” (Kanehisa and Goto, 2000; Yu et al., 2012; Consortium, 2017). Disease Ontology (DO) enrichment analyses and DisGeNET (DGN) enrichment analyses were implemented by the “DOSE” packages in R (Yu et al., 2015).

### Protein–Protein Interaction Network Establishment and Module Analysis

To explore the interaction between hyper-down genes, we uploaded these genes to the STRING database^2^ to get the interaction relationship information between genes, and the cutoff value was set to 0.4. Then, the interactive information was imported into Cytoscape to construct protein–protein interaction (PPI) network diagram (Shannon et al., 2003). Modular analysis using Molecular Complex Detection (MCODE) plugin in Cytoscape with threshold nodes numbers > 4, *k*-score = 2 and MCODE scores > 3 (Bader and Hogue, 2003).

### Machine Learning Algorithm Target Recognition

To minimize the risk of bias in potential target predictions, we used three machine learning algorithms to screen for key characteristic genes that are distinguishable from psoriasis and normal samples. The least absolute shrinkage and selection operator (LASSO) performed by the R package, named “glmnet,” was a regression analysis method that uses regularization to reduce the prediction error (Tibshirani, 2011). Random forest algorithm and support vector machine (SVM) algorithm were both supervised learning methods. Random forest algorithm could generate one decision tree forest, and then through 10-fold cross-validation method to screen out characteristic genes (Rodriguez-Galiano et al., 2012), we used the SVM-recursive feature elimination (SVM-RFE) method to identify the most suitable characteristic genes (Huang et al., 2014). Finally, the genes analyzed by each kind of algorithm were intersected. These overlap genes would be the core genes screened by the final machine learning algorithm. The reliability of these gene predictions would be verified in the external GSE41662 dataset.

### Statistical Analysis

Statistical analyses were conducted using R (version 4.0.3) and Python (version 3.8.2) developed by Guido van Rossum in the Netherlands. The accuracy of predicted potential target genes was judged by receiver operating characteristic (ROC) curve analysis, *p* < 0.05 were regarded statistically significant.

## Results

### General Characteristics of Different Analyses of Different Datasets

To identify the DEGs in the tissues of psoriasis patients compared with matched normal samples, one microarray dataset (GSE30999) had been analyzed and identified 1,589 significant DEGs in psoriasis lesions compared with matched normal skin, in which 634 genes are downregulated and 955 genes are upregulated.

**FIGURE 2:**
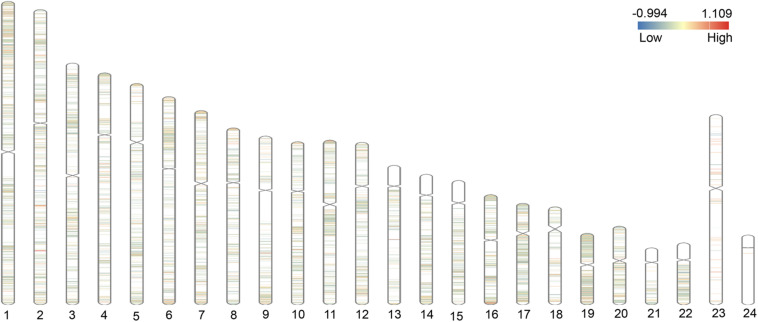
Distribution of differential methylated CpGs (DMCs) on chromosomes. The figure shows the distribution of differentially methylated genes (DMGs) on 22 autosomes, X and Y chromosomes. The red area represents the hypermethylated region, and the blue area represents the hypomethylated region.

**FIGURE 3:**
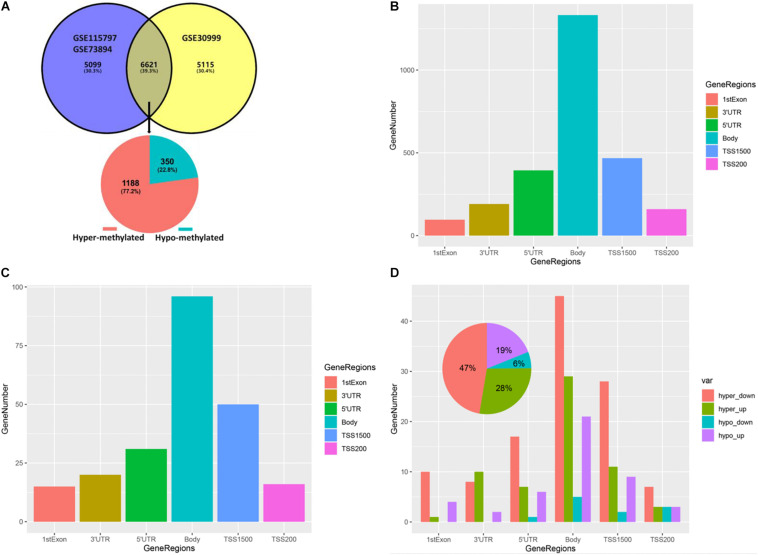
Gene methylation status and DMCs distribution in different gene regions. **(A)** Venn plot of DEGs in dataset GSE30999 and DMGs in the combined GSE115797 and GSE73894 datasets. **(B)** Bar plot of DMCs in different regions of DMGs. **(C)** Bar plot of DMCs in different regions of DEMGs. **(D)** Bar plot of DMCs in different regions of four differentially expressed-methylated genes (DEMGs) groups.

We integrated and corrected the methylation datasets (GSE115797 and GSE73894) and the density plots before and after batch correction were shown in Supplementary Figure 1. We found that DMCs were not distributed on the short arms of chromosomes 13, 14, 15, 21, and 22, as shown in Figure 2. A total of 3,265 DMCs were identified by analyzing the DNAm microarray of psoriasis skin tissue (GSE115797 and GSE73894). Through analysis, it could be seen that 666 DMCs were hypomethylated, and 2,599 DMCs were hypermethylated. In addition, we found that 2,406 DMGs were identified, of which 1,953 genes were hypermethylated and 569 genes were hypomethylated. Similarly, the 1,188 hypermethylated genes accounted for 77.2% of all methylated genes in DMEGs (Figure 3A). The details of the different analysis results of different datasets were shown in Supplementary Table 1.

By analyzing the distribution of DMCs in different gene regions in DMGs and DEMGs, it was found that the distribution of DMCs in different gene regions was mainly concentrated in the body, as shown in Figures 3B,C. Then, we divided DEMGs into hypomethylated and upregulated groups (hypo-up), hypermethylated and downregulated groups (hyper-down), hypomethylated and downregulated groups (hypo-down), and hypermethylated and upregulated groups (hyper-up) according to the gene methylation level and expression level. Finally, the distribution of DMCs in different gene regions in each group was analyzed, which was similar to the above analysis results, as shown in Figure 3D. We also found that almost all DMCs accounted for the largest proportion in each gene region were hyper-down genes, and these 97 hyper-down genes were the highest proportion accounting for 47% of the four kinds of DEMGs, this revealed the importance of hyper-down genes in epigenetic regulation of psoriasis (Figure 3D and Supplementary Table 1).

**FIGURE 4:**
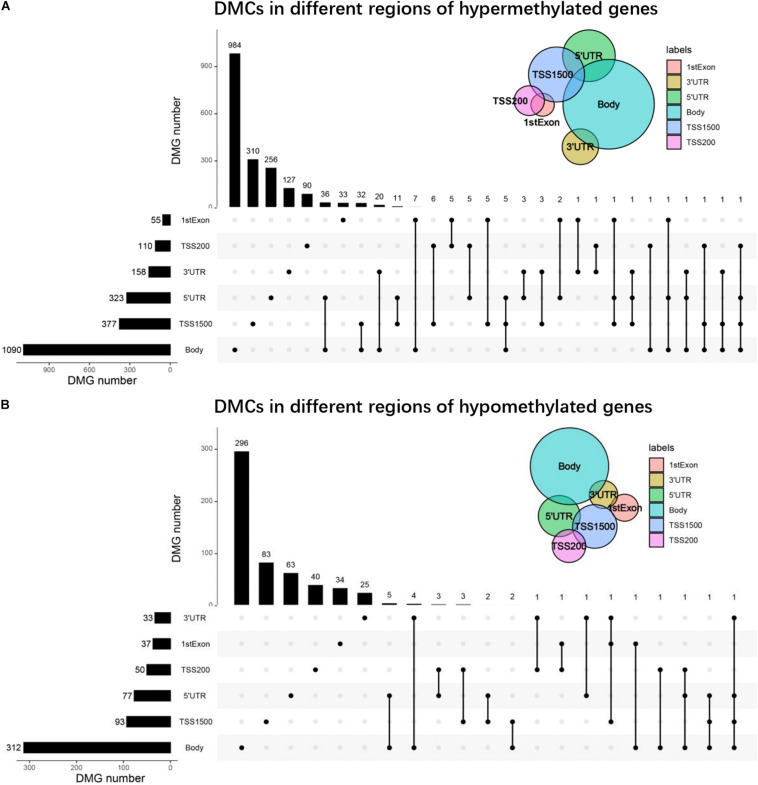
UpSet plots of DMCs. **(A)** The UpSet plot of DMCs in different regions of hypermethylated genes. **(B)** The UpSet plot of DMCs in different regions of hypomethylated genes.

Through the above analysis, we found that each gene region had DMCs, but it was not clear whether there were DMCs in multiple regions of one gene. Therefore, we found that more than 90% of genes were single-gene region methylated by drawing the UpSet maps, no matter hypermethylated genes or hypomethylated genes, and the main focuses were on TSS200, TSS1500, body, 3′UTR, or 5′UTR. About 1% of the genes were polygenic region methylation, for example, there were four gene-region methylations in one gene of hypermethylated genes and hypomethylated genes, respectively (Figures 4A,B).

### Immune Infiltration Analysis of Hypermethylated Genes

**FIGURE 5:**
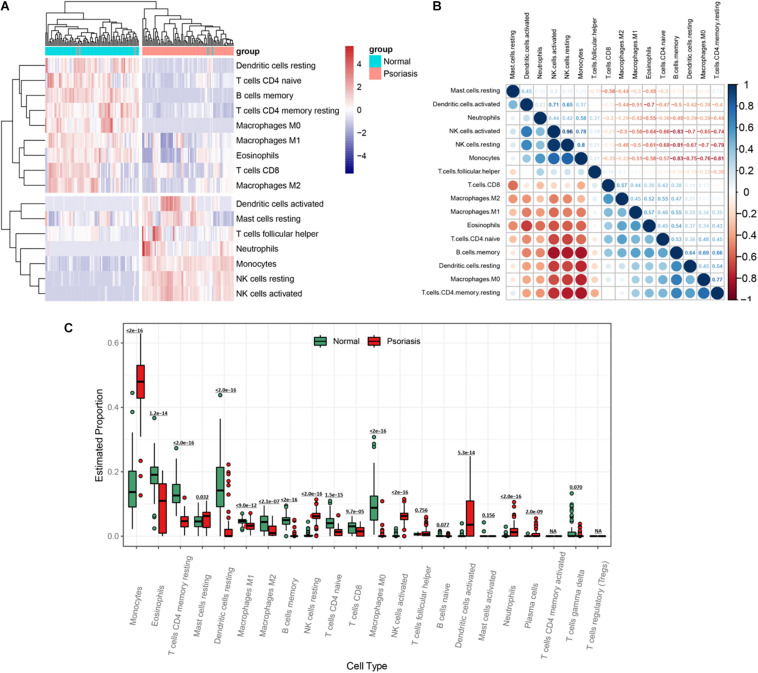
Visualization of immune infiltration analysis of hypermethylated genes. **(A)** Heatmap of immune cell infiltration between psoriasis tissues and normal skin. **(B)** Heatmap of the relationship between different types of immune cells. The strength of the correlation is represented by the dot size and the color depth. The direction of the correlation is represented by the color, red represents negative correlation, and blue represents positive correlation. The darker the color, the stronger the correlation. **(C)** A box plot of the proportion of different types of immune cells between psoriasis tissues and normal skin.

The hypermethylated genes accounted for 77.2% of all methylated genes in DEMGs (Figure 3A), which were used for immune cell infiltration analysis. Through cluster analysis, we found that psoriasis skin tissues were mainly composed of activated dendritic cells (DCs), resting mast cells (MCs), T follicular helper (cTfh) cells, neutrophils, monocytes, resting natural killer (NK) cells, and activated NK cells compared with normal skin tissues (Figure 5A). The correlation of the different types of immune cells was calculated (Figure 5B). We found considerable differences of immune cell composition between psoriatic skin and the normal group (non-lesional skin). Results revealed that M0, M1, and M2 macrophages, eosinophils, resting memory CD4 T cells, resting DCs, naive CD4 T cells, and CD8 T cells were significantly decreased in psoriatic skin, while plasma cells, resting MCs, resting memory CD4 T cells, monocytes, naive and memory B cells, resting MCs, resting NK cells, activated NK cells, activated DCs, neutrophils, and plasma cells were notably increased (Figure 5C).

### Functional Enrichment Analysis of Hyper-Down Genes

**FIGURE 6:**
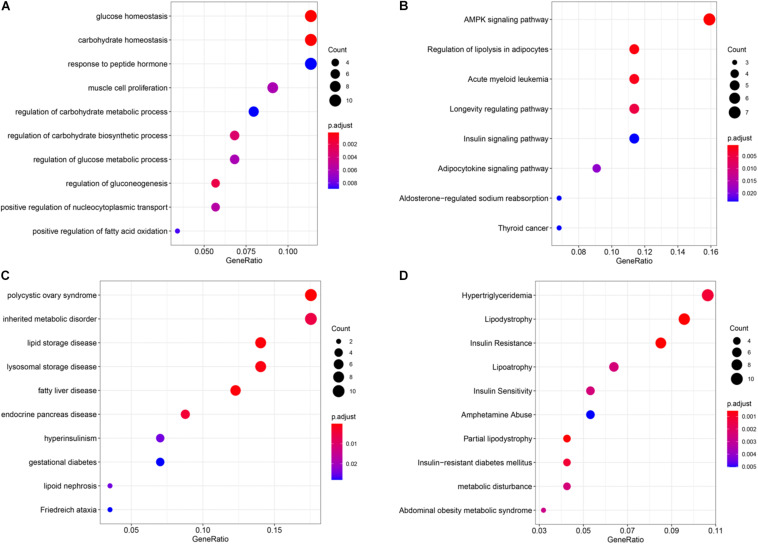
Enrichment analysis of the hyper-down genes. **(A)** Gene ontology (GO) functional enrichment analysis. **(B)** Kyoto Encyclopedia of Genes and Genomes (KEGG) enrichment analysis. **(C)** Disease Ontology (DO) enrichment analysis. **(D)** DisGeNET (DGN) enrichment analysis.

Through enrichment analysis of hyper-down genes, we learned the relationship between these genes and functions, pathways, and diseases. These genes were enriched in the function of glucose homeostasis and carbohydrate homeostasis (Figure 6A). The main pathways were the AMP-activated protein kinase (AMPK) signaling pathway and the regulation of lipolysis in adipocytes (Figure 6B). The diseases were mainly enriched in partial lipodystrophy, insulin resistance, polycystic ovary syndrome, and lipid storage disease (Figures 6C,D). Interestingly, these enrichment results were all related to metabolism.

### Protein–Protein Interaction Network Establishment and Module Identification

**FIGURE 7:**
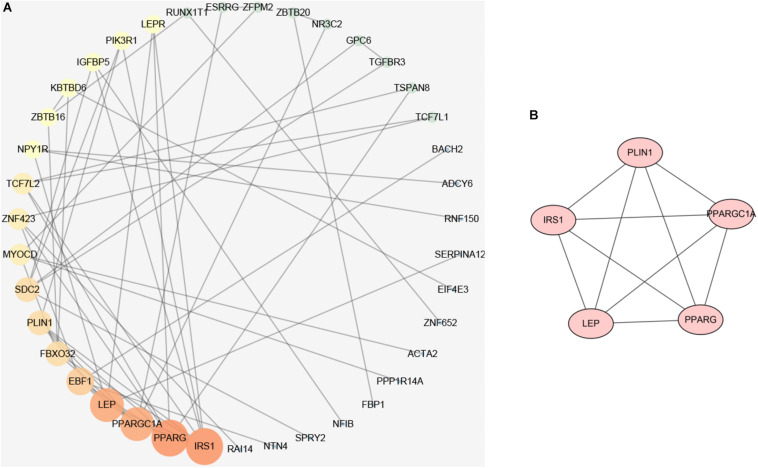
Protein–protein interaction (PPI) network establishment and module analysis of the hyper-down genes. **(A)** PPI network of 97 hyper-down genes. **(B)** Module network with MCODE = 3.3.

To elucidate the interaction between hyper-down genes, Cytoscape visualized the STRING-based PPI network for hyper-down genes. By analyzing 97 hyper-down genes, we got a network interaction graph with 43 nodes and 60 edges, where nodes represented genes, edges represented connections between two genes, and degree value represented the strength of association between genes (Figure 7A). More precisely, the top 10 hub genes of hyper-down genes were insulin receptor substrate 1 (IRS1), PPARG, PPARGC1A, LEP, EBF1, FBXO32, PLIN1, SDC2, MYOCD, and ZNF423 (Figure 7A). One module was identified by MCODE arithmetic (Figure 7B).

### Identification and Validation of Potential Gene Targets

**FIGURE 8:**
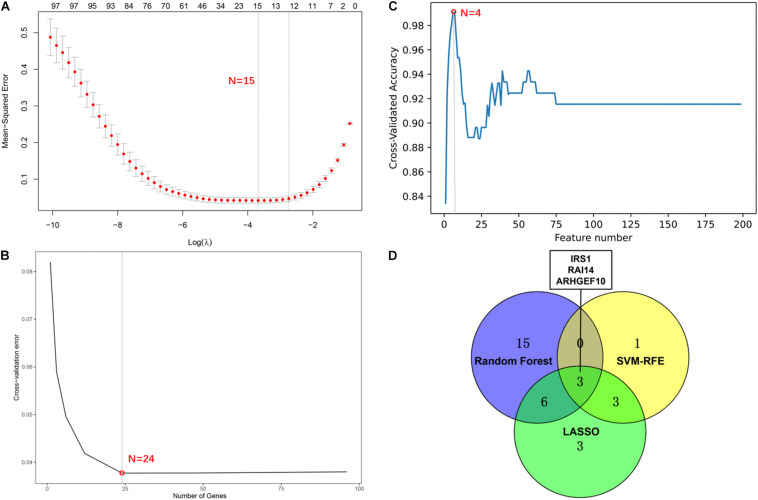
Screening of potential gene targets. **(A)** Least absolute shrinkage and selection operator (LASSO) model to screen gene targets. **(B)** Random Forest model algorithm to screen diagnostic markers. **(C)** Support vector machine-recursive feature elimination (SVM-RFE) algorithm to screen gene targets. **(D)** The venn diagram shows the intersection results of genes screened by three machine learning algorithms.

We screened 15 candidate genes through the LASSO regression algorithm (Figure 8A) and selected 24 candidate genes through the random forest model algorithm (Figure 8B). Four key genes were identified by the SVM-RFE algorithm (Figure 8C). Then, the potential target genes obtained by the three algorithms were intersected, and finally, three overlapping genes (IRS1, RAI14, and ARHGEF10) were obtained (Figure 8D).

**FIGURE 9:**
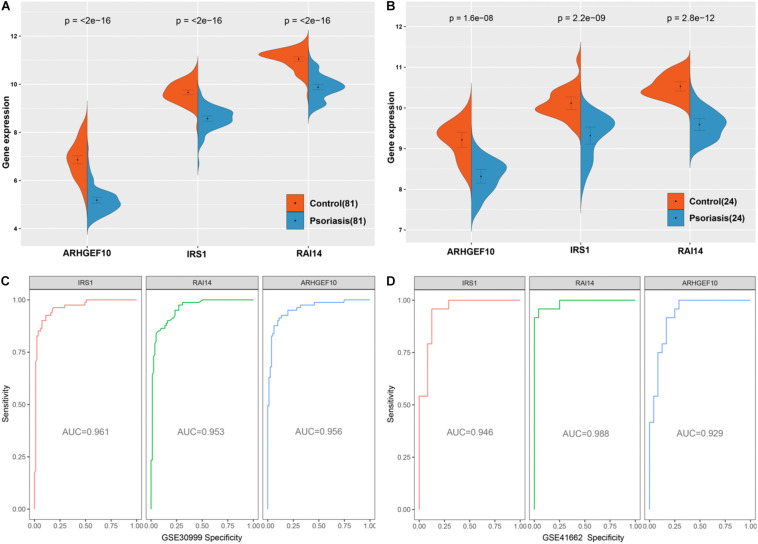
Validation of diagnostic markers. **(A)** The three markers are downregulated in gene set GSE30999. **(B)** The three markers are downregulated in gene set GSE41662. **(C)** ROC curves analysis of gene set GSE30999. **(D)** ROC curves analysis of gene set GSE41662.

The three genes were analyzed in the datasets GSE41662 and GSE30999 and found that the expression levels of IRS1, RAI14, and ARHGEF10 were lower in psoriasis skin lesions compared with the normal control group (Figures 9A,B). The ROC curve analysis in the dataset GSE30999 found that the area under the curve (AUC) of IRS1 was 0.961, the AUC of RAI14 was 0.953, and the AUC of ARHGEF10 was 0.956, as shown in Figure 9C. The ROC curve analysis results in the dataset GSE41662 showed that the AUC of IRS1 was 0.961, the AUC of RAI14 was 0.953, and the AUC of ARHGEF10 was 0.956, as shown in Figure 9D, indicating that the three biomarkers had high reliability.

## Discussion

Identifying epigenetic regulation patterns and certain biomarkers from skin tissues would help diagnose, treat, monitor psoriasis, and help clarify the pathogenesis of psoriasis. In this study, we found that hypermethylated genes accounted for 77.2% of all methylated genes in DEMGs. Therefore, the hypermethylation state of genes in psoriasis lesions was the main methylation state. At the same time, we analyzed and quantified the proportion of immune cells in psoriasis lesions and adjacent normal tissues through the expression of these hypermethylated genes in DEMGs. Through further analysis, we found that 97 hyper-down genes accounted for 47% of the four types of genes and showed the pathways and diseases rich in these genes. About 97 hyper-down genes were constructed by the PPI network and filtered by machine learning algorithms; the intersection was obtained, and new biomarkers that could be regarded as a treatment for psoriasis were obtained. To verify the results of bioinformatics data analysis, we used the external dataset to verify the expression level of these genes.

We could know from the chromosomal distribution map that DMCs were more or less distributed on every chromosome, which revealed that the methylation regulation was very important pathogenesis of psoriasis. We also observed that hyper-down genes played major roles in the epigenetic regulation of psoriasis, which was consistent with the studies of Roberson et al. and Zhou et al. that sites with inverse correlations between methylation and nearby gene expression and were important discriminators of psoriasis (Roberson et al., 2012; Zhang et al., 2013; Zhou et al., 2016; Verma et al., 2018). However, in this study, DMCs were also observed on the sex chromosomes, this might indicate a gender difference in the incidence of psoriasis. A cross-sectional study in Sweden measured the PASI scores of 5,438 patients with psoriasis and showed that there were differences in the degree of psoriasis lesions between men and women (Hägg et al., 2017). Another study also showed that men had predominance over women, and gender differences might be caused by race, living habits, social taboos, etc. (Iskandar et al., 2021), so these differences might also cause differences in DNAm on sex chromosomes (Higgins, 2010; Parisi et al., 2013).

This research has found that almost over half of DMCs in DMGs and DEMGs were concentrated in TSS1500 and body regions. When we divided DEMGs into two groups according to the level of gene methylation, we found that the psoriasis lesions were mainly characterized by hypermethylated genes, which was in line with the findings of recent studies (Zhang et al., 2010; Mervis and McGee, 2020). The DEMGs were further divided into four groups and found that hyper-down genes were the main methylation genes in the development of psoriasis.

The types of immune cell infiltration calculated by the expression of DMEGs hypermethylation genes in psoriasis tissues mainly included CD4 T cells, NK cells, DCs, and so on. CD4 T cells were key components of the immune system and had been shown to play an important role in the pathogenesis of psoriasis (Fry et al., 2015). Recent studies have found that the CD4 T cell subset circulating cTfh cells were related to the development of many diseases including psoriasis (Ma and Deenick, 2014). The cTfh cells were activated in psoriasis with the expression of ICOS, Ki-67, PD-1, and HLA-DR at higher levels and increased production of interleukin (IL)-21, IL-17, and interferon (IFN)-γ. The ratio of cTfh cells and the production of cytokines were significantly reduced after 4 weeks of treatment (Wang et al., 2016). NK cells were well known for their dual functions of cytotoxicity and immune regulation. Studies had shown that more NK cells were infiltrated in the lesions of psoriasis (Son et al., 2009; Zeng et al., 2013; Polese et al., 2020). NKG2C was an active receptor of NK cells and may bind to HLA-E. The NK cells with high expression of NKG2C killed autoreactive T cells and respond to virus-infected cells, which might have an impact on psoriasis prevention (Patel et al., 2013). The inactivation and activation of DCs was an important mechanism to maintain a moderate immune response, which not only played a role in maintaining psoriasis inflammation but also participated in the initiation of inflammation (Wang and Bai, 2020; Novoszel et al., 2021). The neutrophils/lymphocyte ratio and neutrophils activity were significantly increased in patients with psoriasis compared with healthy controls (Wang and Jin, 2020). During the pathogenesis of psoriasis, neutrophils mainly produced IL-17A, which was an important cytokine leading to psoriatic dermatitis (Katayama, 2018). Monocytes (MONs) were pro-inflammatory in psoriasis and were participants in active and dynamic cytokine-mediated signaling (Golden et al., 2021). MCs were important immune cells for the establishment of adaptive and innate immunity, and MC microvesicle interactions might also play a promoting role in the development of psoriasis (Shefler et al., 2014). Therefore, the immune infiltration analysis of hypermethylated genes might provide a new perspective for the study of psoriasis.

The DO and DGN enrichment analyses suggested hyper-down genes, mainly enriched in polycystic ovary syndrome, inherited metabolic disorder, hypertriglyceridemia, lipodystrophy, etc. These diseases are related to metabolism. A previous meta-analysis including 12 studies, either cross-sectional or case–control, indicated a strong connection between metabolic syndrome and psoriasis (OR: 2.26, 95%CI: 1.70–3.01) (Singh et al., 2016). An update of this meta-analytic study including 6 cross-sectional studies and 11 case–control studies found that the association between psoriasis and metabolic syndrome, high blood pressure (OR range: 1.2–2.6), elevated plasma glucose levels (OR range: 1.2–4.6), and a higher prevalence of abdominal obesity (OR range: 2.1–3.8) was noted in patients with psoriasis compared with non-psoriatic controls (Armstrong et al., 2013). A recent meta-analysis of the association between metabolic syndrome and psoriasis included 63 studies (including 15,939 patients with psoriasis and 103,984 controls) and found that 30.29% of patients with psoriasis reported having metabolic syndrome, compared with 21.70% in the control group. The prevalence of metabolic syndrome was increased in patients with psoriasis (OR: 2.077; 95% CI: 1.84–2.34) (Choudhary et al., 2020).

Studies had suggested that the comorbidity of psoriasis and metabolic diseases were due to the excessive production of pro-inflammatory mediators by psoriasis skin lesions [including IL-6, IL-1, IL-23, IL-22, IL-17, vascular endothelial growth factor (VEGF), tumor necrosis factor (TNF)-α, etc.], which could migrate to the systemic circulation, potentially inducing circulatory endothelial dysfunction, increased angiogenesis, systemic insulin resistance, hypercoagulability, and increased oxidative stress. These pathological conditions would in turn induce the occurrence of psoriasis (Arican et al., 2005; Canavese et al., 2010; Lynde et al., 2014; Luan et al., 2015; Gisondi et al., 2018; Martinez-Moreno et al., 2020).

Further KEGG and GO analyses showed that hyper-down genes were mainly enriched in the AMPK signaling pathway and glucose homeostasis, which might be the cause of the comorbidity of psoriasis and metabolic diseases. AMPK, a key sensor of the energy state of all eukaryotic cells, occurred in the form of heterotrimers that catalyze α subunits and regulate β and γ subunits (Carling et al., 2012; Oakhill et al., 2012; Hardie, 2014). Once activated, AMPK restored energy homeostasis by promoting catabolic pathways, leading to ATP production, and inhibiting anabolic pathways that consume ATP (Hardie et al., 2016). In addition to maintaining intracellular energy balance, AMPK also regulated systemic energy metabolism (Hardie et al., 2016; Carling, 2017). Given its key role in controlling energy homeostasis, which was believed to be an important factor in driving changes in a variety of human diseases, AMPK has attracted wide attention as a potential therapeutic target for metabolic diseases, such as type 2 diabetes, obesity, and cancer (Cool et al., 2006; Giordanetto and Karis, 2012; Xiao et al., 2013; Carling, 2017).

A recent study had shown that metformin prevented and treated psoriasis by inhibiting IL-1β targeting AMPK in keratinocytes (Tsuji et al., 2020). Immunosuppressant methotrexate (MTX) had been widely used in the treatment of psoriasis vulgaris (Cribbs et al., 2015; Gladman, 2017), which returned to the normal function of peripheral blood regulatory T cells in plaque psoriasis *via* the AMPK/CD73/mTOR pathway (Yan et al., 2018). Liraglutide is an antidiabetic drug, a glucagon-like peptide-1 (GLP-1) analog, that blocks keratinocyte inflammatory signals by activating AMPK and inhibiting macrophage migration and is considered as a new treatment approach for psoriasis (Yang et al., 2019). Therefore, DNAm in skin tissues affects metabolic function mainly through the AMPK signaling pathway, leading to psoriasis-like lesions. Similarly, improving metabolic function by regulating the AMPK signaling pathway can also be used in the treatment of psoriasis.

We found that through the establishment of a PPI network and machine learning algorithm screening for 97 hyper-down genes, IRS1 was not only one of the three core genes obtained by the machine learning algorithm but also the highest degree value of the PPI network. Insulin could promote wound healing (Goren et al., 2006, 2009), so insulin resistance was not conducive to wound healing. The epidermal changes caused by psoriasis (such as dysdifferentiation and hyperplasia) were consistent with the process of wound healing (Boehncke et al., 2012), so insulin resistance seemed to be an important perspective for studying comorbidities of psoriasis (Brazzelli et al., 2021). IL-1β, a pro-inflammatory cytokine, could induce insulin resistance in psoriasis (Boehncke et al., 2012; Coban et al., 2016).

Aganirsen is an antisense oligonucleotide that can inhibit keratinocyte proliferation and imiquimod induced psoriasis-like dermatitis *via* IRS1 Ser312 and dephosphorylation of Tyr612 in keratinocytes, upregulate the expression of IRS1 and GLUT2 proteins in the human hepatocarcinoma cell line (HepG2), and improve insulin resistance (Li et al., 2019; Xuguang et al., 2019).

Although we had identified potential targets using gene expression and DNAm datasets and explored the pathogenesis of psoriasis, there were two limitations to be considered. First, it was only a study of publicly available data, the biological functions of some related target genes should be explored and verified. Second, even if we integrated datasets of the same platform and selected the ComBat method to remove the batch effect between the two datasets, which could reduce the potential heterogeneity, but some clinical covariates, such as infections, diet, obesity, medications, psychological factors, etc., contributed to the psoriasis process, which could increase the potential heterogeneity (Kaushik and Lebwohl, 2019; Armstrong and Read, 2020).

## Data Availability Statement

The original contributions presented in the study are included in the article/Supplementary Material, further inquiries can be directed to the corresponding author/s.

## Author Contributions

YL conceived and designed the workflow. SC and YL analyzed the work and wrote the manuscript. XY and YL gave analysis advice and created and modified the figures. JS and YL revised the manuscript. All authors approved the manuscript.

## Conflict of Interest

The authors declare that the research was conducted in the absence of any commercial or financial relationships that could be construed as a potential conflict of interest.

## Publisher’s Note

All claims expressed in this article are solely those of the authors and do not necessarily represent those of their affiliated organizations, or those of the publisher, the editors and the reviewers. Any product that may be evaluated in this article, or claim that may be made by its manufacturer, is not guaranteed or endorsed by the publisher.

## Footnotes

1
https://cibersortx.stanford.edu/

2
https://string-db.org/

## Supplementary Material

Supplementary Figure 1 Density plots before and after sample correction. **(A)** Density plot before standardization and removal of batches. **(B)** Density plot after standardization and removal of batches.

Supplementary Table 1 Identification of potential biomarkers for psoriasis using integrated expression and methylation data.
