# Supplementary figures and images for: Identification of Potential Biomarkers for Psoriasis by DNA Methylation and Gene Expression Datasets
Source: Front Genet. 2021 Aug 26;12:722803. doi: 10.3389/fgene.2021.722803 (PMC8427602; doi:10.3389/fgene.2021.722803)

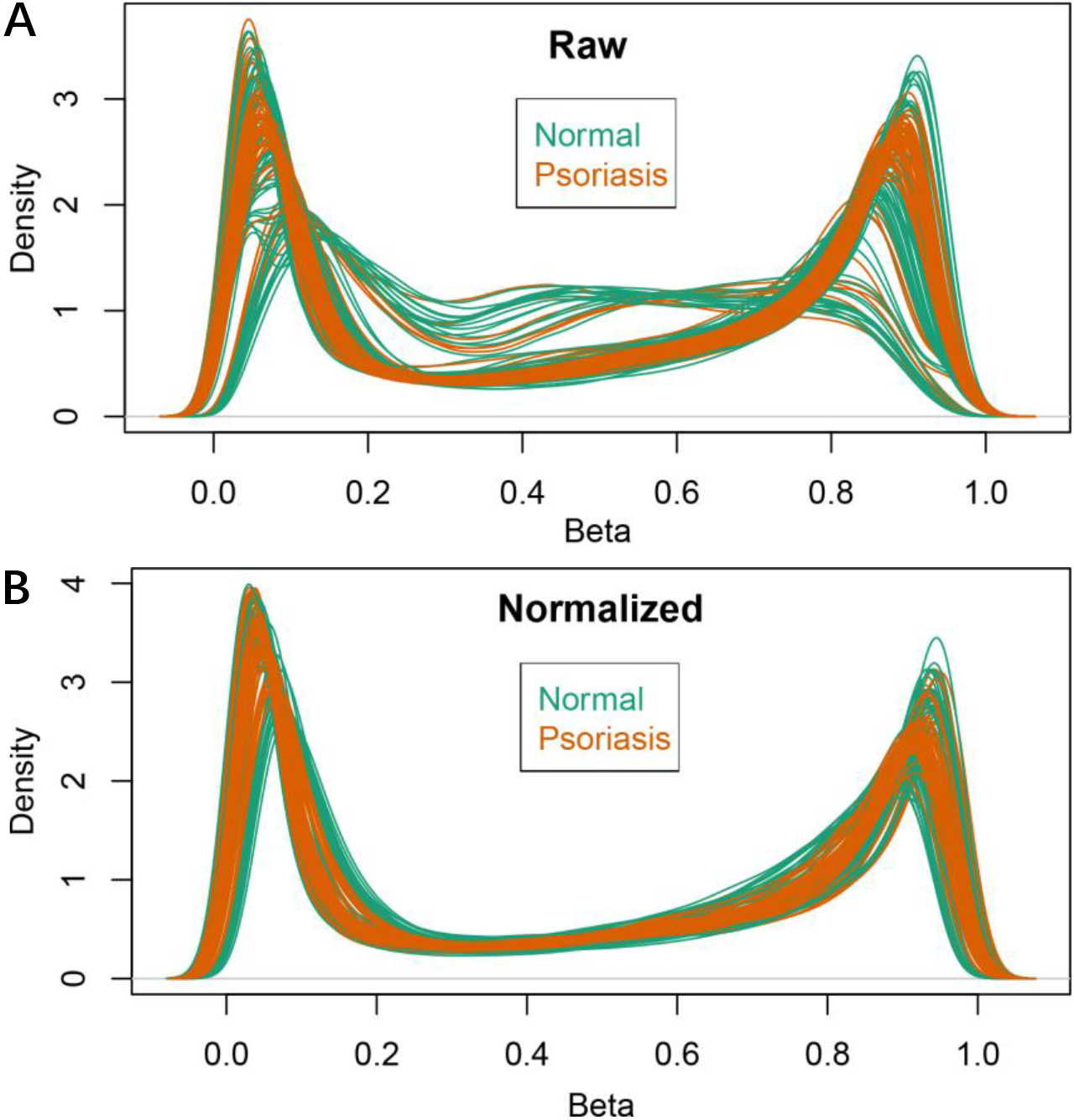

Supplement: Supplementary Figure 1 — Density plots before and after sample correction. (A) Density plot before standardization and removal of batches. (B) Density plot after standardization and removal of batches. [file Image_1.TIF]
